# Effects of Stimulus Temperature and Skin Hydration Levels on Wetness Perception at the Underarm

**DOI:** 10.1111/srt.70170

**Published:** 2025-05-14

**Authors:** Jade Ward, Emilio Verucchi, Dave Swaile, Katie Parker, Peter R. Worsley, Davide Filingeri

**Affiliations:** ^1^ ThermosenseLab Skin Sensing Research Group School of Health Sciences The University of Southampton Southampton UK; ^2^ Procter & Gamble Cincinnati Ohio USA; ^3^ PressureLab Skin Sensing Research Group School of Health Sciences The University of Southampton Southampton UK

**Keywords:** axilla, overhydration, skin, thermal sensation, wetness perception

## Abstract

**Background:**

Experiencing wetness on the skin because of sweating or contact with fluids can induce thermal discomfort. Millions of people apply antiperspirant deodorant products to the underarm to minimise this negative experience. However, the mechanisms underpinning wetness perception at the underarm and the influence of underlying stratum corneum hydration remain under investigation. We aimed to evaluate the role of stimulus temperature and skin hydration levels on wetness perception at the underarm in young participants.

**Materials and Methods:**

Ten healthy participants (5 M/5 F; 29 ± 7 years) underwent a quantitative sensory test during which they reported the perceived magnitude of wetness perception from a short‐duration static application of a cold‐wet (i.e., 5°C below local skin temperature), neutral‐wet (i.e., equal to local skin temperature) and warm‐wet (i.e., 5°C above local skin temperature) stimuli. Wetness perception was assessed on a 100‐mm visual analogue scale (0 = dry; 100 = completely wet), with a repeated measures design exploring the effects of overhydration (+21 %) and dehydration (−40 %) of the underarm's skin.

**Results:**

Our results indicated a higher wetness perception (*p* = 0.012) during the cold‐wet (40 mm, 95 % CI: 25, 56) than during the warm‐wet (25 mm, 95 % CI: 12, 39), and neutral‐wet stimuli (24 mm, 95 % CI: 7, 40). Furthermore, overhydration of the underarm’ stratum corneum can lead to an increase in wetness perceptions upon contact with cold‐wet stimuli only (mean increase: 20 mm, 95 % CI: 3, 36; *p *= 0.024; corresponding to 20 % increase).

**Conclusion:**

Our findings provide novel fundamental insights into the underarm's perceptual responses to wetness, which could inform understanding of the determinants of wet feel associated with periods of sweating and the application of antiperspirant products.

## 1 Introduction

Experiencing wetness on the skin as a result of sweating or contact with fluids has been repeatedly shown to induce thermal discomfort, which is a critical trigger of behavioural responses in humans [1, 2]. Consider, for example, the common experience of wetness at the underarm resulting from thermal or psychogenic sweating. Millions of people apply antiperspirant deodorant products to the underarm on a daily basis to minimise this negative experience [3]. Antiperspirant deodorants are consumer goods hygiene products applied on the underarm to fight odour, reduce sweating and increase self‐confidence [4]. Inefficiencies of antiperspirant action or a lack of application can create visual sweat stains or noticeably wet skin, all of which may negatively impact a person's quality of life [5]. Sweat is secreted at the underarm through three different sweat glands, eccrine, apocrine and apoeccrine [6, 7]. The fluid secreted from each type of sweat gland varies based on its functional properties. The apoeccrine and eccrine glands secrete a watery‐like fluid, whereas, the apocrine glands produce a viscous, odourless lipid‐rich fluid, which, bacteria residing at the underarm react with, resulting in malodour [8]. Daily application of antiperspirant deodorant can minimise sweat secretion and consequently, the malodour [4].

Antiperspirant deodorants have become well‐established worldwide and are available in many physical forms and chemical formulations, such as roll‐on, aerosols, sticks and gels [9]. Whilst generally effective in limiting sweat‐induced wetness at the underarm [4], these products can trigger undesired wetness perceptions at the point of application, that is, when a ‘wet’ product at room temperature (∼23°C) contacts the skin of the underarm (which has a likely temperature of ≥34°C). This ‘acute’ wetness experience may negatively impact the acceptability and comfort of such products. Hence, an increased understanding of the mechanisms of wetness perception at the underarm upon contact with a wet stimulus could inform the design of more comfortable antiperspirant deodorants.

Extensive psychophysical research in wetness perception has indicated that, due to the likely absence of skin hygroreceptors [10], humans learn to perceive wetness through the integration of thermo‐sensory cues. This is triggered by the heat exchange occurring between the skin and a wet stimulus [11, 12], in combination with mechano‐sensory cues resulting from moisture moving across the skin surface [11, 13 ]. Previous work in this area has relied primarily on the application of temperature‐modulated wet stimuli (e.g., above, below or equal to skin temperature) with equal moisture contents to various thermally sensitive regions of the body such as the torso [11], forehead [14], neck [15], lower back [16], dorsal foot [17] and fingertip [18, 19]. This body of literature has repeatedly demonstrated that a cold‐wet stimulus is perceived as wetter than a warm‐wet one given the same moisture content [11], thereby highlighting the primary role of skin cooling in triggering wetness perceptions.

This observation is particularly relevant in the context of applying a cold‐wet antiperspirant deodorant to the warm skin of the underarm. However, the extent to which the established perceptual mechanisms for wetness sensing observed across the body also apply to the underarm, due to a lack of psychophysical research specifically testing the underarm. Indeed, the underarm is a unique skin site, where the stratum corneum (SC) has reduced barrier function due to personal care regimes, including shaving and antiperspirant deodorant application [20]. Shaving, in particular, is often associated with sensory irritation from skin damage through artificial, premature removal of skin cells, leading to a thicker epidermis [21]. This may impact the skin's thermal and tactile sensitivity and the resulting wetness perceptions.

Furthermore, SC hydration levels vary throughout the day via mechanisms of overhydration (e.g., during sweating) and dehydration, and this may alter skin mechanics, such as elasticity, tactile sensitivity and function [22]. Dead SC cells within the epidermis that are exposed to water solutions, such as during a shower, rehydrate in a two‐stage process, the initial increase within the first few minutes filling the voids within the most superficial regions of the SC, followed by a slower linear process of hydration, which induces structural alterations of the SC and swelling to the corneocytes, leading to increased epidermal hydration and surface area [23, 24]. These water‐related changes in the SC modify the chemical structure and mechanical properties of the skin, which in turn may impact tactile sensitivity. Broader evidence indicates that there is a negative correlation between stratum corneum water content and skin roughness [25]. Indeed, it has been previously reported that hydrated skin decreases the sensation of roughness during the contact of fingertips with textured objects [26]. When considering the relationship between wetness perception and roughness perception, Merrick et al. [27] found a negative correlation whereby rougher surfaces felt drier while smoother surfaces felt wetter. However, further research is required to fully establish how the hydration status of the skin of the underarm, and any potential change in skin surface roughness, may impact local wetness sensitivity on this skin site.

Altogether, the evidence above highlights our limited understanding of the mechanisms underlying wetness perception at the underarm and their variation with changes in the hydration levels of the SC. Therefore, the aim of this study was to investigate the role of stimulus temperature and skin hydration levels on wetness perception at the underarm in healthy young participants. We hypothesised that wetness perceptions will be greater as a result of cold‐wet stimulus application as well as with increases in SC hydration.

## 2 Materials and Methods

### 2.1 Ethical Approval

This study was approved by the University of Southampton Ethics Committee (approval no.73017). All participants provided written informed consent prior to testing. The study conformed to the ethical standards set by the Declaration of Helsinki.

### 2.2 Participants

Ten healthy recreationally active participants (5 M/5 F; age 28.8 ± 7.2 years; height 171.3 ± 9.5 cm; body mass 78.1 ± 18.2 kg; BMI 26.4 ± 4.2 kg/m^2^) were recruited for the study. Priori sample size calculation was performed using an effect size corresponding to *f* = 0.71 [28], combined with an *α* = 0.05 and a *β* (power) = 0.8, determined a minimum sample of seven participants. Ten participants were recruited in case of loss to follow‐up.

The volunteers had no history of sensory‐related disorders nor cardiovascular, neurological, or skin‐related conditions (e.g., eczema). Participants were provided with specific instructions prior to testing, including refraining from applying antiperspirant deodorant, and they were required to shave their underarms 24 h prior to the testing sessions. Female participants also provided self‐reports of the last day of their most recent menstrual cycle in relation to the testing day.

### 2.3 Experimental Design

The study was a single‐blind, randomised cross‐over design. Participants took part in two testing sessions on separate days, separated by a minimum of 24 h, performed under the same thermo‐neutral ambient conditions (25°C and 45 % relative humidity). During the sessions, participants performed a standardised quantitative sensory test [14], which consisted in having to report on a visual analogue scale the magnitude of wetness perception arising from the local application of warm‐, neutral‐ and cold‐wet stimuli to the skin of the underarm. This was performed under baseline conditions as well as following a local overhydration and dehydration protocol of the SC. Biophysical skin assessments were also performed to characterise local skin properties.

The dehydration/overhydration protocols were developed following extensive pilot studies. They consisted of (a) the application of a 50 cm^2^ cotton patch, fully saturated with room‐temperature water (1 mL). The patch was covered and secured to the skin with impermeable adhesive tape to prevent evaporation and left in place for 30 min to achieve local overhydration; (b) the application of calcium‐carbonate powder (0.025 g) on the skin side for 30 min for dehydration. Pilot studies indicated this experimental approach induced changes in local SC hydration of ∼±30% from baseline.

It is important to note that the application of the quantitative sensory test at the underarm under dehydration and overhydration skin states required some adjustment from previous studies to minimise the potential carry‐over effect following wet probe application within the same testing session. As such, the centre of the underarm was partitioned into three areas, each of which was stimulated by a different temperature stimulus (i.e., cold‐wet, 5°C below local skin temperature; neutral‐wet, equal temperature as local skin temperature; and warm‐wet, 5°C above local skin temperature). The application area was randomised between participants to minimise any bias due to skin site effects on temperature‐dependent wetness perceptions. Participants were also blinded to the temperature of the stimuli to limit expectation bias. The same investigator performed all testing.

### 2.4 Experimental Procedures

Upon arrival to the laboratory, participants’ anthropometric measurements were collected. Height was measured on a wall stadiometer and weight on a precision scale (KERN 150K2DL, Balingen, Germany). Participantswere then positioned supine on a therapy bed and the testing sites were marked with a washable marker. Subsequently, participants were familiarised with the quantitative sensory test and visual analogue scales using the forearm as a neutral test site (i.e., the midpoint between the wrist and antecubital fossa). Familiarisation procedures have been extensively reported in our previous paper [14].

Upon completion of the familiarisation phase, testing commenced. First, we collected a series of biophysical skin parameters at the underarm with non‐invasive methods, including assessments of local SC hydration ([29], Corneometer; CM 825, CK Electronics, Germany), and of surface roughness (i.e., Rq = the root mean square variation of the surface height, acquired using an optical coherence tomography scanner (OCT), Vivo sights DX, Michelson Diagnostics, UK). SC hydration was measured at each stimulation site, the Corneometer measures the capacitance of the skin surface through the electromagnetic contact in the probe during the spring displacement and converted into arbitrary units [30, 31]. The higher the water content in the SC, the greater the capacitance, leading to high readings. It is of note that changes in skin temperature may affect the water mobility and dielectric properties in the skin [32]; however, the initial hydration stage is unaffected by temperature, whereas the second stage showed a temperature‐dependent increase above 42°C [24]. When considering the skin temperature data collected during our study, we found minimal variation in this parameter between days and across participants (Visit 1; 34.6 ± 1.52°C, Visit 2; 34.0 ± 1.2°C). Hence, it is unlikely that our measurements of skin hydration would have been impacted by skin temperature. Whereas, surface roughness was measured only at the centre of the underarm. Following these measurements, tactile detection thresholds at the underarm were measured using Von Frey's monofilaments (North Coast Medical, Inc., Morgan Hill, LA, USA) employing the up‐down method [33, 34]. At this point, the quantitative sensory test of wetness perception was performed. This consisted of a 10 s application of a hand‐held temperature‐controllable probe (surface area: 1.32 cm^2^), mounted with a 100% cotton patch wetted with 0.8 mL of water, randomised to one of the three marked areas of the underarm. The area stimulated was dependent on the pre‐determined temperature of the stimulus, which was either cold‐wet (5°C below local skin temperature), neutral‐wet (equal temperature as local skin temperature) or warm‐wet (5°C above local skin temperature). Stimulus’ temperature was relative to local skin temperature, which was assessed using infrared thermometry (Spot IR Thermometer TG54; FLIR Systems, Wilsonville, OR, USA). Upon application of the wet stimulus, participants were verbally encouraged to report their local thermal and wetness perceptions on separate visual analogue scales [i.e., 100‐mm wetness scale with anchor points: Dry (0) to Completely wet (100); and 200‐mm thermal scale with anchor points: Very cold (0), Neutral (100) and Very hot (200)]. The three temperature stimuli were applied in a randomised order with 60 s in between applications.

**FIGURE 1:**
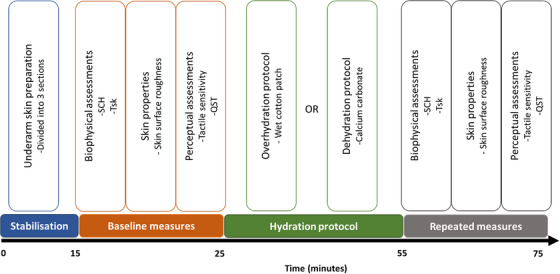
Schematic of experimental design in order of events. SCH, stratum corneum hydration; Tsk, skin temperature; QST, quantitative sensory test.

Upon completion of the baseline quantitative sensory test, and depending on the testing session, the skin of the underarm underwent the 30‐min overhydration or dehydration protocol described in Section 2.3. Upon completion of the protocol, the same sequence of biophysical assessments (i.e., local skin temperature, SC hydration, skin roughness, tactile sensitivity) and the same quantitative sensory test of wetness were performed on the same underarm sites to establish hydration‐dependent changes in wetness perceptions and their biophysical correlates, for a schematic of the experimental procedure see Figure 1.

Data normality and homoscedasticity were assessed using the Shapiro–Wilk and Levene tests, respectively. SC hydration, wetness perception and thermal sensation data were identified to be normally distributed, hence parametric tests were used for analysis. SC hydration, wetness perception and thermal sensation data were analysed separately for the overhydration and dehydration sessions by means of two‐way repeated measures ANOVAs [independent variables: (i) time, with two levels, i.e., prior and following hydration protocol; (ii) skin site for stimulation, with three levels, i.e., warm‐wet site, neutral‐wet site and cold‐wet site)] to determine how skin hydration varied following on each protocol and across testing sites. In the event of a statistically significant main effect or interaction, a post hoc analysis was conducted using a Bonferroni correction. Skin surface roughness data were identified to be normally distributed, paired sample *T*‐test with Bonferroni correction were used to assess overhydration and dehydration outcomes.

Tactile sensitivity data were identified to be non‐normally distributed, thus non‐parametric test were used. Tactile sensitivity data were analysed separately for the overhydration and dehydration sessions by means of Friedman tests followed by [independent variables: (i) time, with two levels, i.e., prior and following hydration protocol; (ii) skin site for stimulation, with three levels, i.e., warm‐wet site, neutral‐wet site and cold‐wet site)] to determine how tactile sensitivity varied following on each protocol and its across testing sites. In the event of a statistically significant main effect, a post‐hoc analysis was conducted using Wilcoxon signed‐ranks tests with a Bonferroni correction.

All data were analysed using SPSS Statistics 19 (version 28.1, Chicago, USA). Statistical significance was set at *p* < 0.05, and data were reported as means, standard deviation (SD) and 95% confidence intervals (CI).

## 3 Results

### 3.1 Stratum Corneum Hydration and Skin Surface Roughness

**FIGURE 2:**
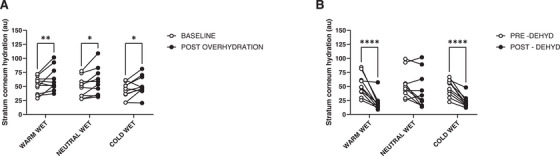
Changes in stratum corneum hydration from baseline to post overhydration (A) and dehydration (B) protocols in the warm‐wet, neutral‐wet and cold‐wet sites at the underarm. Statistically significant differences are pictured (*p < 0.05).

Regarding SC hydration during the overhydration protocol, a statistically significant effect of time (*F* = 10.53, *p* = 0.010), yet no effect of skin site for stimulation (*F* = 3.25, *p* = 0.074) nor an interaction (*F* = 0.92, *p* = 0.883) was found. Specifically, when collapsed over skin site for stimulation, SC hydration increased by 10.0 au (95 % CI: 3.1, 16.7, *p* = 0.010) from a baseline value of 47.6 au (95 % CI: 37.4, 57.8) to a post‐overhydration value of 57.5 au (95 % CI: 43.9, 71.0). This corresponded to an average increase in SC hydration of 21 % (Figure 2A).

During the dehydration protocol, SC hydration significantly changed over time (*F* = 32.34, *p* < 0.001), with no effect of skin site for stimulation (*F* = 3.73, *p* = 0.083), and an interaction effect (*F* = 5.67, *p* = 0.030). When collapsed over skin site for stimulation, SC hydration decreased by 22.4 au (95 % CI: 13.5, 31.4, *p* < 0.001) from a baseline value of 50.9 au (95 % CI: 37.6, 64.2) to a post‐dehydration value of 28.4 au (95 % CI: 17.1, 39.8). This corresponded to an average decrease in SC hydration of 44 % (Figure 2B). Yet, the interaction effect indicated that this average decrease was less pronounced at the neutral‐wet site of stimulation (−10 au, 95 % CI: −23.2, 1.5, *p* = 0.078) than at the warm‐wet (−31.2 au, 95 % CI: −45.6, −16.8, *p* < 0.001) and cold‐wet sites (−25.2 au, 95 % CI: −33.9, −16.5, *p* < 0.001).

Surface roughness had no statistically significant difference between baseline (Rq = 0.021 ± 0.005 µm) and post‐hydration values (Rq = 0.023 ± 0.005 µm; *t* = −0.965, *p* = 0.367) nor dehydration values (Rq = 0.022 ± 0.004 µm; *t* = 0.211, *p* = 0.840).

### 3.2 Wetness Perceptions

**FIGURE 3:**
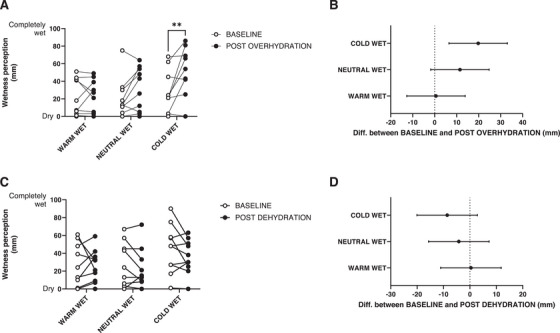
Changes in wetness perception from baseline to post overhydration (A) and dehydration (C) protocols in the warm‐wet, neutral‐wet and cold‐wet sites at the underarm. Statistically significant differences are pictured (*p < 0.05). 95 % confidence intervals for the warm‐wet, neutral‐wet and cold‐wet sites for overhydration (B) and dehydration (D) report. Wetness perceptions were wetter during the cold‐wet stimulation (40 mm, 95% CI: 25, 56) than during the warm‐wet (25 mm, 95% CI: 12, 39), and neutral‐wet stimulation (24 mm, 95% CI: 7, 40) (Figure 3C,D).

Regarding wetness perception data during the overhydration protocol, we found a statistically significant effect of time (*F* = 7.21, *p* = 0.025), of wet stimulus temperature (*F* = 5.16, *p* = 0.020), and an interaction (*F* = 3.63, *p* = 0.047). Specifically, the interaction effect indicated that wetness perceptions increased following the overhydration protocol as a result of the cold‐wet stimulus application only (mean increase: 20 mm, 95 % CI: 3, 36; *p* = 0.024). This corresponded to an average increase in cold‐wet perception of ∼20 % above baseline (Figure 3A,B). Regarding wetness perception data during the dehydration protocol, we found no statistically significant effect of time (*F* = 1.14, *p* = 0.312), a statistically significant effect of wet stimulus temperature (*F* = 6.83, *p* = 0.012), and no interaction (*F* = 1.07, *p* = 0.352). Specifically, when collapsed over time, wetness perceptions were wetter during the cold‐wet stimulation (40 mm, 95% CI: 25, 56) than during the warm‐wet (25 mm, 95% CI: 12, 39), and neutral‐wet stimulation (24 mm, 95% CI: 7, 40) (Figure 3C,D).

### 3.3 Thermal and Tactile Sensations

**FIGURE 4:**
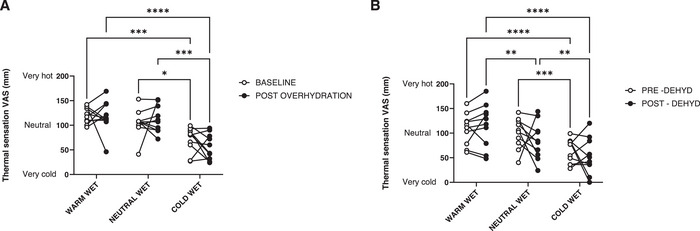
Changes in thermal sensitivity from baseline to post overhydration (A) and dehydration (B) protocols in the warm‐wet, neutral‐wet and cold‐wet sites at the underarm. Statistically significant differences are pictured (*p < 0.05).

Regarding thermal sensation data during the overhydration protocol, we found no statistically significant effect of time (*F* = 0.17, *p* = 0.683), a statistically significant effect of wet stimulus temperature (*F* = 24.94, *p* < 0.001) and no interaction (*F* = 1.15, *p* = 0.339). Specifically, when collapsed over time, thermal sensations were warmer during the warm‐wet stimulation (119 mm, 95 % CI: 107, 131), colder during the cold‐wet stimulation (63 mm, 95 % CI: 49, 78), and close to neutrality during the neutral‐wet stimulation (109 mm, 95 % CI: 95, 123) (Figure 4A). During the dehydration protocol, there was no statistically significant effect of time (*F* = 0.24, *p* = 0.630), a statistically significant effect of wet stimulus temperature (*F* = 13.26, *p* < 0.001), and no interaction (*F* = 2.23, *p* = 0.147). Specifically, when collapsed over time, thermal sensations were warmer during the warm‐wet stimulation (112 mm, 95 % CI: 86, 138), colder during the cold‐wet stimulation (58 mm, 95 % CI: 41, 75), and close to neutrality during the neutral‐wet stimulation (92 mm, 95 % CI: 76, 108) (Figure 4B).

Regarding tactile sensitivity data, we found no statistically significant effect of time nor skin site for stimulation for overhydration (Chi‐square = 1.179, *p* = 0.877) and dehydration (Chi‐square = 7.597, *p* = 0.180).

## 4 Discussion

The study aimed to investigate the role of stimulus temperature and skin hydration levels on wetness perception at the underarm in healthy young participants. We hypothesised that wetness perceptions at the underarm will be greater as a result of cold‐wet stimulus application as well as with increases in SC hydration. Our findings supported both our hypotheses. First, we observed that, whether applied on normo‐, over‐ or de‐hydrated skin, cold‐wet stimuli triggered wetter perceptions at the underarm than warm‐ and neutral‐wet stimuli. Second, we found that an increase in SC hydration of ∼21 % was associated with a ∼20 % increase in wetness perception only when a cold‐wet stimulus was applied on the skin of the underarm. These findings provide novel fundamental insights into the underarm's perceptual responses to wetness, which could inform understanding of the determinants of wet feel associated with the application of antiperspirant products.

Regarding our first observation, our findings are in line with previous research that has indicated that, given the same moisture content, cold‐wet stimuli consistently trigger wetter perceptions than warm‐ and neutral‐wet test conditions [15, 18, 28, 35, 14, 17]. Filingeri et al. [28] have previously reported that a cold‐dry stimulus can elicit a perception of skin wetness, highlighting the importance of skin cooling as a mechanism for the detection of wetness in the absence of a skin hygroreceptor. On the contrary, warmth appears to suppress the perception of wetness when in contact with a wet stimulus [13, 16, 36]. Our current findings confirm that such specific perceptual mechanisms for wetness perception also apply to the skin of the underarm, which is a unique skin site due to its anatomy and biophysical properties resulting from personal care regimes, including shaving and antiperspirant deodorant application [20]. Importantly, our findings extend previous work to demonstrate that the role of coldness in driving wetness perception is maintained irrespective of SC’ hydration levels (i.e., from 44% dehydration to 21% overhydration).

Regarding our second observation, our findings provide novel evidence that an increase in local SC hydration of ∼21 % is associated with an increase in wetness perception during cold‐wet stimulation of the underarm. It has been previously suggested that an increase in water content of the SC could induce swelling of the corneocytes with consequent changes in the mechanical properties of the skin [37] such as friction, pliability and thermal conductivity [38]. It has also been suggested that overhydration may increase skin contact area, in turn leading to an increase in tactile sensitivity [39]. Verrillo et al. [26] also observed that skin dehydration impacted the perception of textured roughness by decreasing tactile sensitivity. Due to the importance of thermal conductivity, tactile sensitivity, and contact area in skin wetness perception, we had expected such mechanisms to underlie the hypothesised increase in wetness perception with overhydration of the SC. Despite not observing any overhydration‐induced changes in skin surface roughness, tactile sensitivity, or thermal sensation at the underarm, the underlying mechanisms for an increase in cold‐wetness perception with overhydration remain unclear. The underarm, being a naturally warm and moist environment that undergoes frequent tactile interactions (such as from clothing and sweat), may be pre‐conditioned, which could reduce the sensitivity to these effects. Additionally, repeated moisture exposure can alter the structural integrity of the corneocytes, impacting their maturity, turnover rate, and thickening of the SC [40]. This adaptation could contribute to the lack of significant biophysical and anatomical changes observed under the overhydration protocol. Further investigation is needed to fully understand these dynamics.

Nevertheless, and from an applied standpoint, these observations are important to drive innovation in antiperspirant deodorant design. Our findings support the view that these products can trigger undesired wetness perceptions at the point of application, that is, when a (cold) ‘wet’ product at room temperature (∼23°C) contacts the skin of the underarm (34.6 ± 1.5°C). Furthermore, our findings indicate that such cold‐wet perceptions may be further increased when antiperspirant application occurs on overhydrated skin. This scenario is commonly associated with the status of the skin following a shower, which is also a common application time for antiperspirant products. Future developments of these products should, therefore, consider approaches to minimise cold‐inducing wetness perceptions upon contact with the overhydrated skin of the underarm, as this scenario is likely to trigger the most intense cold‐wet perceptions.

Whilst the findings of this study have relevant applied implications, there are also some study limitations that are worth highlighting. For example, individual variations in the shape of the underarm may impact specific perceptual outcomes as a result of individual variability in contact area during both hydration manipulations and wet stimulus applications. The chemical composition of fluids in antiperspirants affects their thermal conductivity, potentially altering skin cooling rates upon contact [41]. Skin cooling is known to enhance wetness perception [28]. However, the relationship between a fluid's thermal conductivity and wetness perceptions is not fully understood. Future studies should therefore also consider how the current findings may vary when fluid application extends beyond water to include fluids likely found in the chemical formulations of commercially available antiperspirant deodorants. As these fluids may present varying heat capacity and thermal conductivity properties, it is reasonable to expect that their application may differentially impact wetness perceptions upon contact with the underarm.

## 5 Conclusions

In conclusion, we have demonstrated that the established perceptual mechanisms underlying wetness perception in healthy young adults also apply to the skin of the underarm, as we found that cold‐wet stimuli were consistently reported to be wetter than warm‐ and neutral‐wet ones when applied to either normally hydrated as well as over‐ and dehydrated skin. Furthermore, we provide novel evidence that overhydration of the underarm’ SC can lead to an increase in wetness perceptions upon contact with cold‐wet stimuli. These observations carry applied implications to inform the design of antiperspirant deodorants that minimise wet perceptions upon contact and help improve users’ comfort.

## Conflicts of Interest

The authors declare no conflicts of interest.

## Data Availability

The data will be available upon publication at the University of Southampton data repository (PURE; URL to be activated upon publication).

## References

[1] D. Filingeri and G. Havenith , “Peripheral and Central Determinants of Skin Wetness Sensing in Humans,” Handbook of Clinical Neurology 156 (2018): 83–102. 30454611 10.1016/B978-0-444-63912-7.00005-9

[2] A. P. Gagge , “A New Physiological Variable Associated With Sensible and Insensible Perspiration,” American Journal of Physiology‐Legacy Content 120 (1937): 277–287.

[3] A. Watkinson , R. S. Lee , A. E. Moore , S. E. Paterson , P. Pudney , and A. V. Rawlings , “Is the Axilla a Distinct Skin Phenotype?,” International Journal of Cosmetic Science 29 (2007): 60–60.

[4] P. Teerasumran , E. Velliou , S. Bai , and Q. Cai , “Deodorants and Antiperspirants: New Trends in Their Active Agents and Testing Methods,” International Journal of Cosmetic Science 45, no. 4 (2023): 426–443. 36896776 10.1111/ics.12852 PMC10946881

[5] D. F. Swaile , L. T. Elstun , and K. W. Benzing , “Clinical Studies of Sweat Rate Reduction by an Over‐the‐Counter Soft‐Solid Antiperspirant and Comparison With a Prescription Antiperspirant Product in Male Panelists,” British Journal of Dermatology 166 (2012): 22–26. 10.1111/j.1365-2133.2011.10786.x 22385032

[6] K. Sato , R. Leidal , and F. Sato , “Morphology and Development of an Apoeccrine Sweat Gland in Human Axillae,” American Journal of Physiology 252 (1987): R166–180. 3812728 10.1152/ajpregu.1987.252.1.R166

[7] W. B. Shelley and H. J. Hurley , “The Physiology of the Human Axillary Apocrine Sweat Gland12,” Journal of Investigative Dermatology 20 (1953): 285–297. 13052978 10.1038/jid.1953.35

[8] P. A. Low Chapter 51 ‐ Sweating. in Primer on the Autonomic Nervous System (Third Edition), ed. D. Robertson , I. Biaggioni , G. Burnstock , P. A. Low and J. F. R. Paton (Academic Press, 2012).

[9] A. Benohanian , “Antiperspirants and Deodorants,” Clinics in Dermatology 19 (2001): 398–405. 11535380 10.1016/s0738-081x(01)00192-4

[10] R. Clark and O. G. Edholm , Man and His Thermal Environment (Edward Arnold, 1985).

[11] D. Filingeri , D. Fournet , S. Hodder , and G. Havenith , “Body Mapping of Cutaneous Wetness Perception Across the Human Torso During Thermo‐Neutral and Warm Environmental Exposures,” Journal of Applied Physiology (1985) 117 (2014a): 887–897. 10.1152/japplphysiol.00535.2014 25103965

[12] D. Filingeri , D. Fournet , S. Hodder , and G. Havenith , “Tactile Cues Significantly Modulate the Perception of Sweat‐Induced Skin Wetness Independently of the Level of Physical Skin Wetness,” Journal of Neurophysiology 113 (2015a): 3462–3473. 25878153 10.1152/jn.00141.2015 PMC4455488

[13] D. Filingeri , B. Redortier , S. Hodder , and G. Havenith , “Thermal and Tactile Interactions in the Perception of Local Skin Wetness at Rest and During Exercise in Thermo‐Neutral and Warm Environments,” Neuroscience 258 (2014c): 121–130. 24269934 10.1016/j.neuroscience.2013.11.019

[14] A. Valenza , A. Bianco , and D. Filingeri , “Thermosensory Mapping of Skin Wetness Sensitivity Across the Body of Young Males and Females at Rest and Following Maximal Incremental Running,” Journal of Physiology 597 (2019): 3315–3332. 31093981 10.1113/JP277928

[15] R. Ackerley , H. Olausson , J. Wessberg , and F. Mcglone , “Wetness Perception Across Body Sites,” Neuroscience Letters 522 (2012): 73–77. 22710006 10.1016/j.neulet.2012.06.020

[16] D. Filingeri , B. Redortier , S. Hodder , and G. Havenith , “Warm Temperature Stimulus Suppresses the Perception of Skin Wetness During Initial Contact With a Wet Surface,” Skin Research and Technology 21 (2015c): 9–14. 24612108 10.1111/srt.12148

[17] C. Wildgoose , A. Valenza , A. Buoite Stella , K. Feka , A. Bianco , and D. Filingeri , “Ageing Reduces Skin Wetness Sensitivity Across the Body,” Experimental Physiology 106 (2021): 2434–2444. 34676631 10.1113/EP090027

[18] O. Jay and G. Havenith , “Finger Skin Cooling on Contact With Cold Materials: An Investigation of Male and Female Responses During Short‐Term Exposures With a View on Hand and Finger Size,” European Journal of Applied Physiology 93 (2004): 1–8. 15205959 10.1007/s00421-004-1146-x

[19] C. Merrick , R. Rosati , and D. Filingeri , “Skin Wetness Detection Thresholds and Wetness Magnitude Estimations of the Human Index Fingerpad and Their Modulation by Moisture Temperature,” Journal of Neurophysiology 125 (2021): 1987–1999. 33826451 10.1152/jn.00538.2020 PMC8356767

[20] G. Turner , A. Moore , V. Marti , S. Paterson , and A. James , “Impact of Shaving and Anti‐Perspirant Use on the Axillary Vault,” International Journal of Cosmetic Science 29 (2007): 31–38. 18489309 10.1111/j.1467-2494.2007.00361.x

[21] P. Chaturvedi , W. Kroon , G. Zanelli , and P. R. Worsley , “An Exploratory Study of Structural and Microvascular Changes in the Skin Following Electrical Shaving Using Optical Coherence Topography,” Skin Research and Technology 30 (2024): e13830. 38951871 10.1111/srt.13830 PMC11217022

[22] H. Tagami , “Location‐Related Differences in Structure and Function of the Stratum Corneum With Special Emphasis on Those of the Facial Skin,” International Journal of Cosmetic Science 30 (2008): 413–434. 19099543 10.1111/j.1468-2494.2008.00459.x

[23] L.‐C. Gerhardt , V. Strässle , A. Lenz , N. D. Spencer , and S. Derler , “Influence of Epidermal Hydration on the Friction of Human Skin Against Textiles,” Journal of the Royal Society Interface 5 (2008): 1317–1328. 18331977 10.1098/rsif.2008.0034 PMC2607440

[24] M. Morin , T. Ruzgas , P. Svedenhag , et al., “Skin Hydration Dynamics Investigated by Electrical Impedance Techniques In Vivo and In Vitro,” Scientific Reports 10 (2020): 17218. 33057021 10.1038/s41598-020-73684-y PMC7557913

[25] H. J. Lee , S. R. Park , D. I. Kwon , M. S. Park , and D. H. Lim , “Depth Profiling of Epidermal Hydration Inducing Improvement of Skin Roughness and Elasticity: In Vivo Study by Confocal Raman Spectroscopy,” Journal of Cosmetic Dermatology 21 (2022): 4810–4817. 35073423 10.1111/jocd.14795

[26] R. T. Verrillo , S. J. Bolanowski , C. M. Checkosky , and F. P. McGlone , “Effects of Hydration on Tactile Sensation,” Somatosensory & Motor Research 15 (1998): 93–108. 9730110 10.1080/08990229870826

[27] C. Merrick , R. Rosati , and D. Filingeri , “The Role of Friction on Skin Wetness Perception During Dynamic Interactions Between the Human Index Finger Pad and Materials of Varying Moisture Content,” Journal of Neurophysiology 127 (2022): 725–736. 35044853 10.1152/jn.00382.2021 PMC8897031

[28] D. Filingeri , B. Redortier , S. Hodder , and G. Havenith , “The Role of Decreasing Contact Temperatures and Skin Cooling in the Perception of Skin Wetness,” Neuroscience Letters 551 (2013): 65–69. 23886487 10.1016/j.neulet.2013.07.015

[29] P. Clarys , R. Clijsen , J. Taeymans , and A. O. Barel , “Hydration Measurements of the Stratum Corneum: Comparison Between the Capacitance Method (Digital Version of the Corneometer CM 825) and the Impedance Method (Skicon‐200EX),” Skin Research and Technology 18 (2012): 316–323. 22092664 10.1111/j.1600-0846.2011.00573.x

[30] M. Anthonissen , D. Daly , R. Peeters , et al., “Reliability of Repeated Measurements on Post‐Burn Scars With Corneometer CM 825,” Skin Research and Technology 21 (2015): 302–312. 25382262 10.1111/srt.12193

[31] T. V. A. Westermann , V. R. Viana , C. Berto Junior , C. B. Detoni da Silva , E. L. S. Carvalho , and C. G. Pupe , “Measurement of Skin Hydration With a Portable Device (SkinUp() Beauty Device) and Comparison With the Corneometer(),” Skin Research and Technology 26 (2020): 571–576. 31957168 10.1111/srt.12833

[32] C. Rossmann and D. Haemmerich , “Review of Temperature Dependence of Thermal Properties, Dielectric Properties, and Perfusion of Biological Tissues at Hyperthermic and Ablation Temperatures,” Critical Reviews in Biomedical Engineering 42 (2014): 467–492. 25955712 10.1615/critrevbiomedeng.2015012486 PMC4859435

[33] S. R. Chaplan , F. W. Bach , J. Pogrel , J. Chung , and T. Yaksh , “Quantitative Assessment of Tactile Allodynia in the Rat Paw,” Journal of Neuroscience Methods 53 (1994): 55–63. 7990513 10.1016/0165-0270(94)90144-9

[34] W. Dixon , “The Up‐and‐Down Method for Small Samples,” Journal of the American Statistical Association 60 (1965): 967–978.

[35] D. Filingeri , B. Redortier , S. Hodder , and G. Havenith , “Regional Differences in the Cutaneous Thermal Sensitivity to Wetness Across the Torso (1104.10),” FASEB Journal 28 (2014b).

[36] A. Christogianni , R. Bibb , A. Filtness , and D. Filingeri , “Regional Skin Wetness Perception and Its Modulation by Warm and Cold Whole Body Skin Temperatures in People With Multiple Sclerosis,” American Journal of Physiology‐Regulatory, Integrative and Comparative Physiology 323 (2022): R648–R660. 36036454 10.1152/ajpregu.00149.2022 PMC9602777

[37] R. R. Warner , K. J. Stone , and Y. L. Boissy , “Hydration Disrupts Human Stratum Corneum Ultrastructure,” Journal of Investigative Dermatology 120 (2003): 275–284. 12542533 10.1046/j.1523-1747.2003.12046.x

[38] Z. D. Draelos Chapter 18 ‐ Proper Skin Hydration and Barrier Function. in Nutritional Cosmetics, ed. A. Tabor and R. M. Blair (William Andrew Publishing, 2009).

[39] A. Samadi , T. Yazdanparast , M. Shamsipour , et al., “Stratum Corneum Hydration in Healthy Adult Humans According to the Skin Area, Age and Sex: A Systematic Review and Meta‐Analysis,” Journal of the European Academy of Dermatology and Venereology 36, no. 10 (2022): 1713–1721. 35681001 10.1111/jdv.18297

[40] R. R. Wickett and M. O. Visscher , “Structure and Function of the Epidermal Barrier,” American Journal of Infection Control 34 (2006): S98–S110.

[41] W. M. Bergmann Tiest and A. M. Kappers , “Tactile Perception of Thermal Diffusivity,” Attention, Perception, & Psychophysics 71 (2009): 481–489. 10.3758/APP.71.3.481 19304639

